# Private equity firms and industrial policy: elaborating the state-finance nexus in state-led markets

**DOI:** 10.1080/13563467.2024.2318422

**Published:** 2024-02-22

**Authors:** Imogen T. Liu

**Affiliations:** Department of Political Science and Public Administration, Vrije Universiteit Amsterdam, Amsterdam, the Netherlands

**Keywords:** Private equity, industrial policy, infrastructural power, state capitalism, sovereign wealth funds

## Abstract

Under what conditions can the state discipline private equity firms into delivering the investment required to meet the coming needs of industrial transformation? States have sought to crowd in private capital to finance industrial development, but the results have so far been less than satisfactory. Prevailing accounts of financial industry power largely characterise an arms-length state-finance relationship that has unfolded in private-led markets where private equity firms have contributed to the secular growth in non-productive economic activity. This article problematises the assumption of private-led markets and argues that state-led markets present a counterfactual in which the disbursement of public money entails strict policy discipline and tight embedding between the state and private equity firms, which provides the conditions for them to emerge as unlikely champions of industrial policy. Two cases of co-investment between Chinese and European sovereign wealth funds demonstrate the power dynamics at play. Where PE firms in the Sino-Irish co-investment facilitated the international scaling of Irish firms in China, the PE firms operating in Europe failed to embed Chinese firms into regional supply chains in the Sino-Belgian co-investment.

## Introduction

Market-based forms of state-supported industrial development have in the past few decades given private capital greater steering capacity in OECD economies. In the European Union (EU), industrial policy has aligned with competition policy to promote the competitiveness of domestic industrial firms, coalescing around a set of arms-length policy mechanisms where private capital is looked to as the primary engine of industrial growth (Wigger 2019, Bulfone 2023), giving private equity (PE) and venture capital (VC) firms an essential role in the investment chain between the state and industry.^1^

PE/VC firms represent the infrastructural power of finance vis a vis the state. They have accrued market power as the interests of for-profit business clients have become increasingly central to public investment, calling into question the extent to which the financial industry can be expected to support the industrial transformations being called for in an era of intensifying geoeconomic competition, ecological collapse and global indebtedness (Berry 2020, Weiss and Thurbon 2021, Babić et al. 2022, Gabor and Sylla 2023, Schneider 2023).

Extant accounts of the power of finance largely speak to governing contexts in which the state has acceded market steering capacity to private capital, which has led to the secular growth in non-productive economic activity across OECD economies (Bayliss *et al*. 2023, Gibadullina 2023). This article problematises the assumption of private-led markets and shows how state-led markets where the disbursement of state capital entails strict policy discipline has created the conditions for PE firms to emerge as unlikely champions of industrial policy. Such an approach contributes to debates on the power of finance vis a vis the state (Mertens and Thiemann 2018, Braun 2020, Cooiman 2023) by distinguishing analytically between private and state-led markets and the political entanglements the distinction implies as a structuring condition that can alter the terms of engagement between the state and the financial industry.

This article demonstrates how these dynamics play out in a most similar systems comparison of Sino-European sovereign wealth fund (SWF) co-investment into (1) China’s state-led markets between the China Investment Corporation and the Irish Strategic Investment Fund, and (2) European private-led markets between the China Investment Corporation and Belgium’s Société Fédérale de Participations et d’Investissement/Federale Participatie en Investeringsmaatschappij (SFPIM), or Federal Holding and Investment Company. Originally conceived as vehicles for long-term liquidity, SWFs have increasingly taken on investment mandates that prioritise key sectors like digital and hi-tech, regional infrastructure and the growth of small and medium-sized enterprises in a policy landscape that has favoured market-based industrial policy. As SWFs have followed the industry shift toward unlisted assets, co-investment, where a SWF takes up a minority stake alongside other peer investors in a PE fund, has become the preferred mode of investment into unlisted assets that has seen PE firms become indispensable financial intermediaries of SWFs (IFSWF 2021a, 2021b).

In China’s state-led markets, the infrastructural power of PE firms is ground in the imperative to reap both profits and extra-profit returns. The disbursement of Chinese state capital requires responsiveness to industrial policy. The tight links between the PE firm and the China Investment Corporation made possible in markets that privilege proximity to politics supported the growth of Irish firms in China that was the industrial policy mandate of the Irish Strategic Investment Fund. The inverse dynamic is observed of the Sino-Belgian co-investment. EU markets governed by the regulatory state are characterised by arms-length independence between the state and privately-owned firms. The PE firms appointed to the co-investment were delegated an extra-profit mandate in markets where their infrastructural power derives from their ability to price risk and return on a for-profit basis, demonstrating the mismatch between state mandate and the disciplining imperatives of private-led markets.

These findings are based on ongoing dialogues (Clark 1998) with 14 interviewees between October 2019 and July 2022 in Ireland, Belgium and Beijing including three directors, one analyst at the Irish Strategic Investment Fund; two executives, two PE managers and one financial analyst from the Belgian Federal Holding and Investment Company; one director and one vice president at the China Investment Corporation; two officials from the European Commission; and one academic.^2^ Annual reports, legislation and secondary academic sources triangulate the observations gleaned from interview.

## Theoretical framework

### The power of finance vis a vis the state

In capitalist economies, financial markets are a key social arena that states seek to govern since it is the competitive exchange between firms, banks, investors and individuals that allocates capital as a necessary input of socialised production. In the tradition of Michael Mann’s conceptualisation of infrastructural power (Weiss and Thurbon 2018, Schwartz 2019), when states seek to govern financial markets through direct participation, it results in the creation of interdependent relationships with other market participants, giving them steering power over economic governance across a range of policy fora including industrial policy, monetary policy, development and infrastructure financing (Mertens and Thiemann 2018, Braun 2020, Alami *et al*. 2021).

Extant accounts have shown how the infrastructural power of finance varies across different markets for financial products in which states willingly conform to market logics of risk and return because failing to do so blunts their own power to govern (Braun 2020). VC is one such market in which VC firms have become an industrial policy mechanism to support digital and innovation industry development in the EU, where not only is the allocation of state capital based on VC firms’ determination of the risk-return profile of particular technologies, but the very investment mandates of the EU, i.e. industrial policy goals, have been influenced by the preferences of VC firms (Cooiman 2023).

However, in addition to variation across *financial products*, infrastructural power also varies across markets based on *ownership composition* because the ownership claim on property, whether private or state, entails different sets of political entanglements. In markets where the state has acceded market growth and development to private capital, where growth in economic activity is largely funded by private sources of investment and generated by privately-owned firms, financial market actors are disciplined by the need to maximise risk-adjusted private returns. In contrast, state capital entails both profit maximisation and extra profit returns – social and political – to the state (Lee 2018).

When states seek to govern through private-led markets, leveraging the power of private capital for governance purposes, market failure arguments have been the basis for intervention (Mertens and Thiemann 2018). Stemming from the adoption of nominally Keynesian ideas into mainstream, neoclassical economic policymaking (Crouch 2009, van ‘t Klooster 2022), market failure arguments rest on the assumption that the market, barring short run cyclical downturns, is largely capable of efficient allocation. Private capital, which is needed for productive expansion, is given pride of place in market steering while state capital, along with other forms of active state interventionism, are necessary *only* when the market has failed to effectively allocate private investment, typically in times of weak demand.

By implication, too much public investment is inefficient and distorts the allocative mechanism of the market, which can achieve full employment in the long run. State capital implies a premium that is attributed to the soft-budget constraint. Since state-owned firms can draw on state capital as an unlimited source of funds, they are not proscribed by a hard budget, which implies that state-ownership is less efficient, irrationally risk-taking, and offers less returns compared to private investors, generating an excess burden on taxpayers (Kornai 1986, Lazzarini and Mussachio 2018).

This has resulted in the institutionalisation of a state-finance relationship in private-led markets that is politically arms-length. Public investments are deemed good value and therefore legitimate when there is separation of powers between the state as a regulator and shareholder, and the state as an investor. Independence of operational decision-making across the investment chain is enshrined at different levels of governance, stemming from firm-level corporate governance to supranational rules and regulations (Dixon and Liu 2019, Volberding 2021).

However, private-led markets have failed to coordinate the financing required to address the global underinvestment gap (Bernards 2024). Underinvested sectors often entail long investment horizons, high risks and significant start-up costs – infant industries like clean tech, aviation and biotechnology. Market failure arguments for an industrial policy would suggest that the private returns on these investments is undervalued because their long-term returns to society are not captured in market pricing (Rodrik 2014). This has led to innovative market correcting public investments that ‘de-risk’ the participation of private capital via public guarantees (Gabor and Sylla 2023).

However, such arguments are prone to criticism of the rent seeking behaviour of private capital. The Minsky-Marx critique of ‘Keynesian’ private-led markets is that they lead to high-profit high-investment growth that gives in to the worst impulses of the rentier (Bellafiore 2020). De-risking public investments are only artificially state capitalist since they are intended to induce private investment, as opposed to a socialised public investment; amplifying, as opposed to constraining, the competitive pressures of capital expansion without regard to the limits of the market (Crotty 1993, Clarke 2002).

Where private capital has been in the driver’s seat, social need has been subordinated to social production. The interests of financial market actors are geared toward the direct appropriation of value to deliver short-term gains or, in the case of long investment horizons, steady cashflows as opposed to reinvestment in productive expansion, a dynamic that has been thoroughly documented in the financialisation, rentier capitalism and asset manager capitalism literature (Christophers 2020, Braun 2022, Bayliss *et al*. 2023, Gibadullina 2023, Rogers 2023).

Where state capital has lead market development, the state has had an interest in disciplining financial market actors. The best supporting case has been the development of high-tech industries in the United States, Japan, Korea and Taiwan. Underpinned by large-scale public investments in the form of direct fiscal transfers, preferential government loans, subsidies and tax credits, industry development has reflected the geoeconomic and geopolitical interests of the state (Block 2008, Weiss and Thurbon 2021). The Cold War catalysed the establishment of the Small Business Investment Company in the United States, in which the state provided government-backed loans and equity financing through VC firms (Weiss, 2014), while catch-up development and global competitiveness are often cited motivations behind East Asian developmental states where the provision of state capital has come with strings attached (Yeung 2011).

Perhaps more crucially, the relationship between the state and market actors in state-led markets promises close monitoring, regulation and coordination by a coherent state bureaucracy. Minsky advocated for public investment and public consumption-driven economies because the socialisation of investment gives the state the means to constrain private speculation (Bellofiore 2020). Generous subsidies have been deployed as an industrial policy mechanism in Japan and Korea, but the ‘disciplined support’ of the state has characterised the disbursement of these monies where the metrics against which they are evaluated is policy-based (Weiss 1995, pp. 607–9). In markets where state capital leads growth and development, financial market actors have the potential to be disciplined by the state, which can mobilise financial market actors to direct investment into strategic sectors with long investment horizons, substantial start-up costs and higher risk-return ratios, turning the soft-budget constraint into a soft-budget advantage. State capital has proven particularly crucial in catalysing new industry development, fostering national champions and reorienting production from domestic to international markets, all of which have become increasingly high priority industrial policy strategies. It is against the backdrop of East Asian developmental catch-up in the 1980s and 1990s that strategic investment funds like Singapore’s Temasek and Malaysia’s Khazanah have, through state shareholding, fostered the international competitiveness of domestic state-owned enterprises (Yeung 2011, Dixon 2022).

The terms private-led and state-led are of course used reflexively. State and private ownership do not map conveniently onto territorially-proscribed boundaries. The nature of market development varies across industry and geography in ways that reflect the very different composition of social forces that underpin political economies, but there is analytical value in drawing out the distinction. It problematises the assumption of uniformly private-led markets and forces an articulation of state-led markets as a counterfactual antidote to the failure of private investment in OECD economies. The extent to which financial market actors may be relied on to support the structural transformations necessary to build more resilient economies requires that we account for the nature of market development and how the particular state-finance relationships that characterise those markets shape financial power in the first place. In the next section, I apply this analytical lens to the world of public investment and the PE firm.

## The infrastructural power of PE firms

Two real world economies approximate the ideal type characteristics of the state-PE firm relationship across private – versus state-led markets (Table 1).

**Table 1.** Defining features of the state-PE investment chain in European private-led markets versus Chinese state-led markets.

	EU	China
*State ownership* (% total banking assets)	Low (<15%)	High (>50%)
*Investment logic* and underlying assumptions	Risk-adjusted profit maximisation; long-run efficiency of markets	Extra-profit ‘industrial development’ maximisation; underdeveloped markets require state coordination
Strength of *political connections*	Weak. Arms-length independence	Strong. Institutionalised revolving doors between political office, state bureaucracy and state-owned investors
*Corporate governance* of stateownership	Legal enshrinement of EU competition policy and other rules on non-distortive market behaviour	Legal enshrinement of party state representation
*Investment practices*	Based on deep market knowledge and narrow sectoral/asset class expertise, asymmetric contracts and predatory investment practices maximise non-productive surplus value capture	Geared toward policy responsiveness and political salience (e.g. princeling hires) in addition to market expertise in deal-making; capture of non-productive surplus value contingent on productive investment

For the most part, PE firms have concentrated in the older industrialised economies of Western Europe and North America where the world’s largest, highly competitive financial markets have flourished. Despite the growing adoption of Keynesian ideas in economic policymaking, market development has principally reflected the Smithian faith in free private market exchange. The European regulatory state has overseen a steady pace of privatisation. In the EU, *state ownership* accounts for less than 15 per cent of all banking assets (EBRD 2020, pp. 70–2).

The dominance of private capital has materialised in a certain *investment logic* that has rendered PE firms essential to the public investment chain and weakened the *political connection* between the state and PE firms. Underpinned by rational choice theories like the efficient markets hypothesis and modern portfolio theory, it has become widely accepted that arms-length financial intermediation is the preferred form of investment management, which minimises transaction costs and maximises risk-adjusted profits (Clarke and Monk 2017). Backed by EU competition policy on state aid, merger control, anti-trust and other rules governing non-distortive market behaviour that have been adopted in the *corporate governance* mandates of state-owned investors, European SWFs, state-owned investment banks and funds delegate investment management to independent PE managers, giving PE firms power over asset allocation (Bachher *et al*. 2016, Mertens and Thiemann 2018, Cooiman 2023).

PE firms have developed *investment practices* over time that consolidate their ability to extract profits along the investment chain that constitutes a form of non-productive surplus value appropriation (Froud and Williams 2007, Morgan and Nasir 2021). The sectoral and asset-class expertise of PE/VC managers is paramount in deal-making, reflecting the need for deep knowledge of the highly competitive start-up, early and growth stage market space (Kuebart 2019, Cooiman 2023). The contracts which govern the relationship between state-owned investors and PE firms is asymmetrical. State-owned investors by definition are asset owners, for they have a legal claim to invest the assets under management. Asset owners contract out investment management to PE and VC firms who place assets in the market on behalf of their clients (Clark and Monk 2017). The investment management agreements that have become standard in the industry may be used to hold owners hostage where they lack the knowledge to write contracts in their favour. The fee-based model of value generation prevalent in PE exposes asset owners to greater risk than PE firms, encouraging speculative investment while they can continue to charge management fees (Christophers 2015).

In contrast to the EU, financial markets in China have only emerged in the past two decades with the privatisation and corporatisation of state-owned enterprises, the entry of China to the World Trade Organisation, and the advent of domestic stock exchanges (Chiu and Lewis 2006, Walter and Howie 2012). The state has taken a strong hand in creating, but also stabilising and correcting markets, resulting in gradual financial liberalisation and a supporting role for private capital in driving the direction of financial market development (Petry 2020). Despite steady increase in the number of privately-owned firms between 2010 and 2022, *state ownership* still accounts for over 50 per cent of China’s banking assets (EBRD, 2020).

The ability of PE firms to initiate and control the public investment chain requires *political connections* because the boundaries between the state and the market are more porous. The revolving doors between administrative, party and corporate appointments are institutionalised. The central organisation department of the state is responsible for major executive appointments of China’s leading state-owned enterprises where it has become increasingly common to make joint appointments for the top positions and rotate the leadership among state-owned enterprises (Leutert 2018). Party representation within state-owned enterprises is moreover enshrined in state owned *corporate governance* mandates. With the securitisation of finance in recent years against the backdrop of intensifying US–China rivalry, PE firms and the state have only become more interlinked (Pearson *et al*. 2022).

The infrastructural power of PE firms in China stems from their ability to be politically resourced, that is, adopt extra-profit *investment logics*. The state has guided the formation of regional cross-border financial networks that has attracted a high concentration of financial intermediaries to service the growing flows of capital in and out of China (Töpfer 2018; Gemici and Lai 2020). The world’s leading asset managers and investment banks including Morgan Stanley and BlackRock have been enlisted to internationalise Chinese state-owned enterprises and facilitate the investment of state capital into strategic assets (Liu and Dixon 2021). They have reaped profits in China but on the proviso that their strategies have aligned with the developmental prerogatives of the state to leverage finance for industrial expansion.

The most successful managers are those that are able to respond to policy developments and leverage their proximity to politics. Princeling hires, the children of political elites, has become accepted *investment practice* in PE and investment banking, giving PE firms and investment banks access to both the networks of the political establishment and of global finance (Robertson 2015; Forsythe *et al*. 2019). By implication, their embedding in China’s state-led markets is a source of value in deal-making with international investors.

## SWF co-investment and PE power

**Figure 1.**
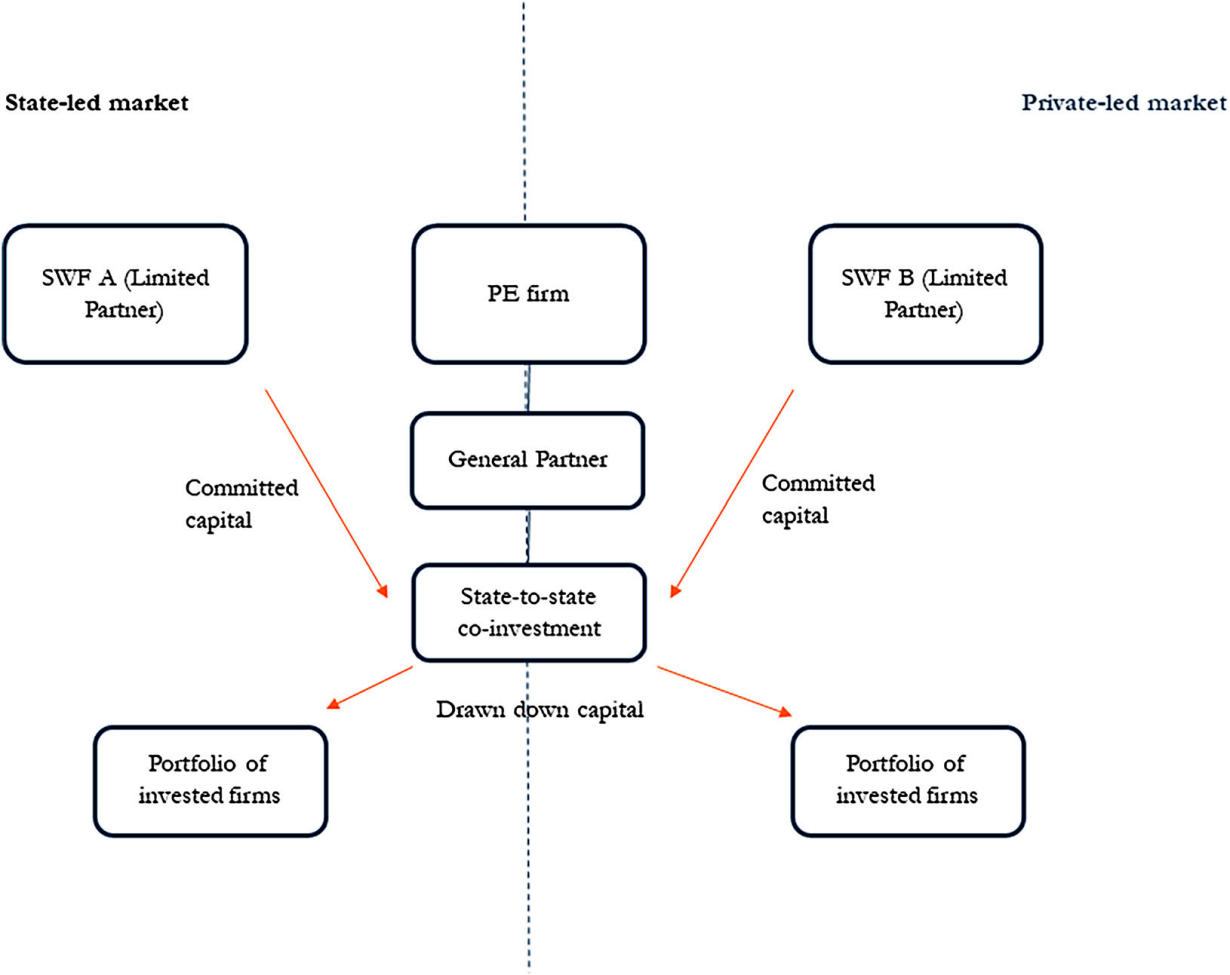
The SWFs-PE firm co-investment chain. *Source: Author’s own design.*

SWF co-investment presents the perfect opportunity to observe how the infrastructural power of PE firms operates differently across private and state-led markets since they invest transnationally, reflected in the low spatial proximity of PE (and VC) (Fritsch and Schilder 2008). Figure 1 explains the co-investment dynamics.

PE firms take on an essential role in identifying, allocating and managing assets on behalf of their SWF clients. Co-investments are typically set up as limited liability partnerships with a general partner and limited partners. The PE firm provides the general partner, the PE manager, to manage a given fund while the SWF typically commits an amount of capital (committed capital) as a limited partner with no managerial oversight over the portfolio of invested firms. The general partner then sources the portfolio of companies that will draw down the fund.

The general partner is a gatekeeper for the allocation of state capital to invested firms. While both PE firms and their state-sponsored clients are governed by the conventions of investment management, namely limits on managerial control of limited partners over invested firms, SWFs are beholden to mandates set by their sponsor, the state. This is reflected in the mandate of the PE fund, typically set up with a predefined focus, whether it is industry, product or region specific. In the case of the European Investment Fund, it took on a mandate from the German Ministry for Economic Affairs targeting German technology-focused VCs, which has become a major VC market in recent years (Cooiman 2023).

Crucially, the relationship between the SWFs and the PE firm is transactional. The PE firm is delegated governance authority to manage state capital and therefore power along the investment chain that can be exploited to extract private profits. This relationship of dependency accords with prevailing conceptualisations of infrastructural power in private-led markets where independence of investment decision-making has been institutionalised. However, in state-led markets there is an added caveat that stems from PE firms’ proximity to politics. Strict policy discipline is a condition of their ability to extract private profits along the investment chain. PE firms are incentivised to seek profits *and* extra-profit returns on state capital investment. In the context of market-based industrial policy, this entails the coupling of domestic firms to global production networks.

Two cases of co-investment illustrate how the infrastructural power of PE firms plays out differently across European private versus Chinese state-led markets. The first is the China-Ireland Technology Growth Fund established by the China Investment Corporation and Irish Strategic Investment Fund. A PE firm called WestSummit with strong links in Ireland and China played a decisive role in fulfilling a key Irish investment mandate, supporting the international expansion of Irish small and medium-sized enterprises in China. The second case is the Spiegelfonds established by the China Investment Corporation and the Belgian Federal Holding and Investment Company, where a series of PE firms that operated across European and Chinese markets were not politically resourced in European private-led markets in the same way as WestSummit was in China.

These cases are symmetrical insofar as the real-world messiness of many variables, small number of cases allows in a most similar comparison. First, both the Irish Strategic Investment Fund and the Belgian Federal and Holding Company must comply with the separation of powers that extends from EU regulation where independence of commercial decision-making must be established in the case of state-owned enterprises (Svetlicinii 2021). Both are global SWFs that adhere to investment management convention. As small-scale SWFs, they do not have the scale to manage investments in-house, preferring instead to outsource to external managers which has granted PE firms discretion over European state capital.

Second, they are of comparable size. In 2020, the Irish Strategic Investment Fund maintained a EUR 12.7 billion combined portfolio, 3.4 billion of which is at its discretion to invest in global markets (NTMA 2021). The Belgian Federal Holding and Investment Company had a combined portfolio of over EUR 14 billion including delegated assignments from the federal state and EUR 759 million of discretionary capital at its disposal (SFPIM 2021). While on the face of it, the Irish Strategic Investment Fund appears significantly larger, the difference pales in comparison to the major SWFs like the China Investment Corporation (USD 1.3 trillion) and Norges Bank (USD 1.38 trillion) (CIC 2023, Norges Bank 2023).

Third, they are sponsored by the states of Ireland and Belgium, two small open economies. Their respective governments have delegated their SWFs market-based industrial policy mandates. The Irish Strategic Investment Fund was established in 2011 with a domestic development mandate where a certain proportion of all investments were to go to Irish companies, either scaling indigenous firms or supporting regional employment (NTMA 2014). Similarly, the Belgian Federal Holding and Investment Company which was established in 2006 after a series of state-owned consolidations created a SWF with more capital and discretion to serve industrial development at the federal level (SFPIM 2020). On the decision of its governing board, the SWF has committed to invest almost exclusively in Belgian small and medium-sized enterprises. In the co-investment with the China Investment Corporation, a certain proportion of investments must go toward Belgian companies (SFPIM 2021).

Finally, the China Investment Corporation, established in 2007, is the world’s second largest SWF with USD 1.2 trillion under management that seeks both globally diversified returns and investments that align with China’s industrial policy goals in energy, infrastructure, digital and innovation. Co-investments in particular were intended as a gateway for invested Chinese firms into European markets (Haberly 2011, Liu and Dixon 2021).

### The China-Ireland technology growth fund

**Figure 2.**
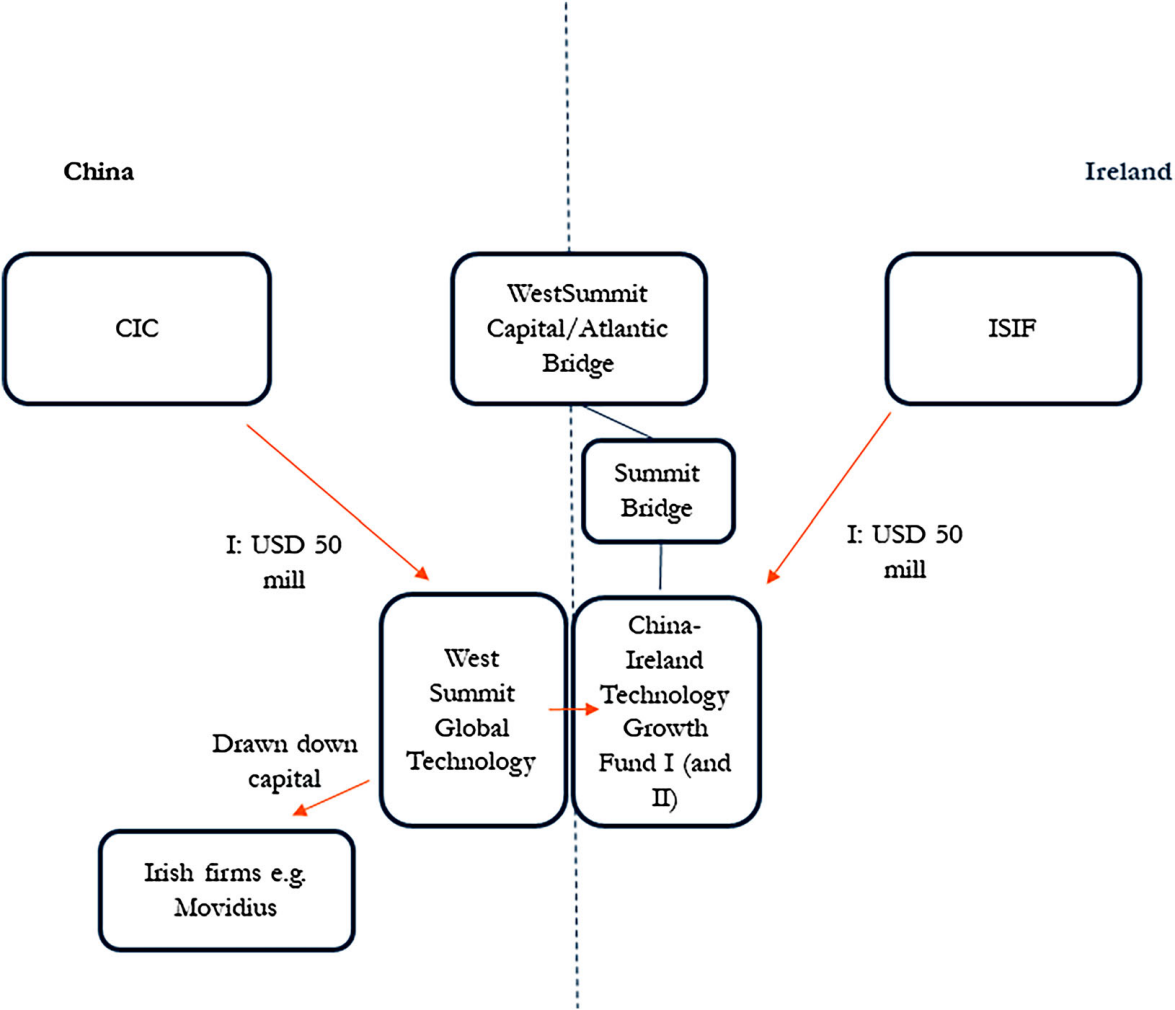
The China Ireland Growth Tech Fund I investment chain. *Source: Author’s own design.*

The China-Ireland Growth Tech Fund I (herein Fund I) is a VC fund launched in 2013 by the National Pension Reserve Fund, the predecessor to the Irish Strategic Investment Fund, and the China Investment Corporation targeting growth stage Irish companies in core technology sectors with a strategic ambition to access the Chinese market or Chinese companies seeking access to the European single market by locating in Ireland. The fund was intended to function as an international scale-up VC, less specialised in geography or industry unlike growth focused VCs which tend to be narrowly framed around specific technologies in digital and health (Kuebart 2019). Fund I had an initial 50–50 commitment of USD 100 million that is now fully invested. Figure 2 maps the key relationships.

There was initial interest from both the National Pension Reserve Fund and the China Investment Corporation in a co-investment. The former had wanted to attract VC capital to Ireland in the wake of the financial crisis that could go toward scaling Irish firms. The latter was interested in Ireland as a key node in technology supply chains into the EU. A memorandum of understanding was signed in Beijing in 2012 in a high-level meeting where both prime ministers were present (ISIF 2014).

Concurrent to these high-level discussions were the operational negotiations between the Irish Strategic Investment Fund and a VC firm, WestSummit, that drove the deal from here on out. WestSummit is a transnational PE/VC firm that specialises in growth stage VC technology with offices across Beijing, Silicon Valley and later Dublin.

Three features characterise WestSummit’s infrastructural power in the co-investment. First, its power is a product of its tight embedding in China’s state-led markets that materialised in the close relationship it had with the China Investment Corporation. As a central-level state-owned investor with ownership stakes in the four major banks, the China Investment Corporation is a key node in China’s financial and production networks. China Investment Corporation did not want to invest directly into a VC fund that it could not scale and had made it a condition that WestSummit come on board as general partner.^3^ Headed by a founding partner that was a former China Investment Corporation employee, WestSummit emerged out of the SWF and continues to be China Investment Corporation’s principal VC manager. The familiarity with which the founding partner of WestSummit engaged all the senior CIC investment managers attests to the strength of political connections.^4^

Second, and as a result of its political linkages, WestSummit could control the investment chain between the SWFs and invested firms, which it used to extract private profits. Fund I’s asset allocation was made by the two PE firms involved, and not the SWFs. At the time WestSummit did not have a presence in Ireland and the Irish Strategic Investment Fund had made the introduction to Atlantic Bridge, an Irish growth-stage VC firm that the Irish Strategic Investment Fund had a strong relationship with. Atlantic Bridge was also based in Silicon Valley and Dublin. WestSummit and Atlantic Bridge subsequently became co-general partners of Fund I through a joint venture called SummitBridge. Despite the original mandate of Fund I, which was to invest in both Chinese firms expanding into the EU via Ireland, and Irish firms hoping to scale up into the Chinese market, SummitBridge, especially WestSummit, had judged there to be good growth opportunities in China, resulting in the first round (Fund I) investing in Irish technology firms’ Chinese expansion, as opposed to Chinese firms’ European expansion.^5^ Moreover, WestSummit had wanted to diversify its investor base and had made it a condition of a potential co-investment that the SWFs commit to a separate technology fund with WestSummit that would not be confined by geography, in other words, to invest for profit. WestSummit leveraged its infrastructural power in the deal to diversify its own investor base.

Third, at the same time that WestSummit could extract private profits, its centrality in Chinese financial and production networks was also used to achieve the extra-profit interests of the Irish state to scale up the invested Irish firms in China. In the decade since establishment, the joint venture SummitBridge had made a number of investments in Irish VCs expanding into the Chinese market, one of the most successful being a company called Movidius, an Irish chip company that had raised USD 90 million in funding between 2006 and 2016. Through WestSummit’s connections, Movidius quickly expanded into China and became a major chip provider of the Shenzhen-based robotics company, DJI (Schroth 2016).^6^ Modivius was subsequently acquired by Intel, becoming Intel Movidius.

For the Ireland Strategic Investment Fund whose primary interest in Fund I was to scale Irish firms, WestSummit exercised infrastructural power to deliver on its industrial policy mandate. The access to domestic firms and supply chains that WestSummit could secure within China’s state-led markets was the primary rationale for the ultimate success of Fund I. The establishment of a second fund, the China Ireland Growth Tech Fund II launched in 2018, was driven in large part by the earlier successes of Fund I, and was intended as a reciprocal fund, to invest state capital in Chinese firms’ productive expansion in Europe, but the progress of Fund II has been stalled due to Covid-19 (NTMA 2021).

### The Spiegelfonds

**Figure 3.**
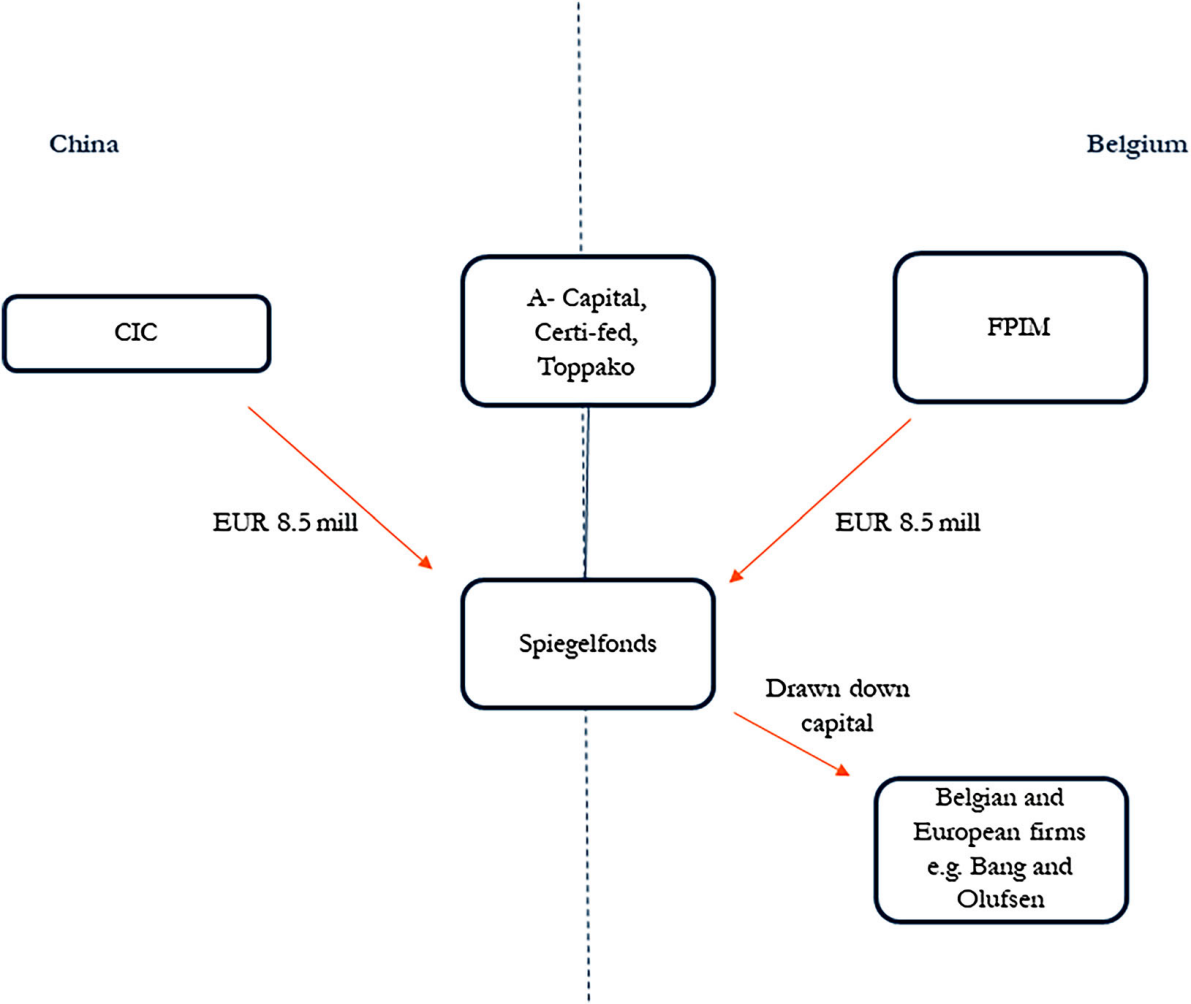
The Spiegelfonds co-investment chain. *Source: Author’s own design.*

The Spiegelfonds demonstrates the tension between the nature of infrastructural power in private-led markets and the industrial policy mandate delegated to the PE funds. The Spiegelfonds was designed to mirror an earlier highly successful Renminbi-denominated fund called the China Belgium Direct Equity Investment Fund in a reciprocal arrangement by investing in European, but particularly Belgian, and Chinese companies throughout the EU. For the China Investment Corporation, the Spiegelfonds was intended as a means to invest state capital into European brands, technologies and distribution channels via Brussels that had strong growth potential in China, and that could be a means of coupling Chinese production to European supply chains. Established in 2012 with a 50–50 commitment from both SWFs totalling EUR 17 million, the Spiegelfonds was fully invested with two out of four projects of Belgian origin (SFPIM 2021). Figure 3 illustrates the co-investment dynamics.

The Spiegelfonds highlights the tension between the nature of PE power in private-led markets and the policy discipline demanded of them in order to serve as industrial policy mechanisms. First, they were not politically resourced like WestSummit was in China’s state-led markets that is the basis for strict policy discipline. Established as a public utility company under Belgian law, the governance charter of the Belgian Federal Holding and Investment Company takes as a template the OECD Guidelines on Corporate Governance of State-Owned Enterprises (OECD 2015) which emphasise independence of state-owned enterprise operational autonomy from the government (Guideline II, p. 18), and fair competition principles that specify ‘[state-owned enterprises] relations with all financial institutions, as well as non-financial state-owned enterprises, should be based on purely commercial grounds’ (Guideline III, p. 20). The Belgian Federal Holding and Investment Company is furthermore expected to observe all general laws and regulations, including EU competition rules that proscribe interventions that deviate from those of (private) market competitors and in this way ensure a level playing field in the Single Market (SFPIM 2020).^7^

Second, the PE firms could not leverage the co-investment to extract private profits. A crucial condition that an infrastructural power approach to finance articulates is that market actors are able to exploit the relationship of dependence with the state to appropriate additional non-productive surplus, terms that would be necessary for them, as for-profit entities, to have the equivalent of a soft budget advantage in order to make extra-profit investments. Unlike WestSummit, which could dictate within the terms of the co-investment the creation of a separate fund that constituted an extra revenue stream, the PE firms operating in Europe were not politically resourced in the same way. One telling indication of the arms-length relationship between the SWF clients and PE firms was the high turnover rate of PE firms appointed to the Spiegelfonds. The first, A Capital, was a PE firm focused on European midcap growth firms, co-investing with Chinese investors into leading European midcap firms. With offices in Beijing, Brussels and Hong Kong, it was well placed to manage the co-investment, but the investments it sourced did poorly and A Capital went out of business in 2019. Based on shareholder decision, Certi-Fed, a subsidiary of the Belgian Federal Holding and Investment Company, temporarily took over as general partner until June 2020 when a third firm, Toppako Capital, was named as general partner at the onset of the Covid-19 pandemic (SFPIM 2021).

Third and by extension, the PE firms were operating in European PE markets where their governance value derives from their ability to accurately price risk and return. In PE/VC this translates into practices that leverage their deep supply chain knowledge. In contrast to the Sino-Irish co-investment, where the lack of sectoral expertise was overcome by political proximity, the lack of focus appeared to work against the PE firms in securing access for Chinese firms to European supply chains. Although all the Spiegelfonds investments were in technology firms, they were in a broad range of sectors, including Epigan, a Belgian semiconductor company, Sunpartners Technology, a Belgian solar energy technology firm, and Bang and Olufsen, the Danish audio-technical equipment specialist. In 2019, Epigan was sold to SOITEC, an established French semi-conductor company, while an offer by an undisclosed Chinese company that was 25 per cent higher was rejected by Epigan’s Board of Directors.^8^ Although the sale was profitable, and the Spiegelfonds still maintains a share in Epigan, the investment did little to further supply chain embedding for Chinese semi-conductor companies, a situation that is only likely to be exacerbated given the subsequent politicisation of Chinese investment in Europe. Following a similar logic, Sunpartners Technology went into liquidation, precluding further expansion involving Chinese firms (SFPIM 2021).

The Spiegelfonds’ experience with general partners demonstrates the tensions between the for-profit nature of the PE firms’ infrastructural power in European private-led markets, and the extra profit mandate delegated by the China Investment Corporation to further Chinese supply chain embedding in Europe. By contrast, the success of the Sino-Irish co-investment, which was due in large part to the embedding of WestSummit in China’s state-led markets, were a source of infrastructural power that the Irish Strategic Investment Fund could draw on to scale Irish small and medium-sized enterprises in China.

## Conclusion

A simple question lies at the heart of this article: under what conditions can the state discipline PE firms into delivering the investment required to meet the coming needs of industrial transformation? There is untapped potential in the power of PE firms that can be leveraged to achieve industrial policies. Extant debates have highlighted the inherent risks in governing through financial markets (Mertens and Thiemann 2018, Wigger 2019, Braun 2020, Cooiman 2023) and the difficulties in taming the rentierism of private capital for public purpose (Christophers 2020), but China’s state-led markets offer a real world counterfactual that demonstrates how states can alter the terms of engagement with PE firms, which exemplify the unproductive rentier, to emerge as unlikely champions of industrial policy.

The findings would seem to imply that what is needed is a return to big state spending in which investment necessary for productive growth is socialised, as opposed to merely a supporting character that boosts private investment in times of weak demand. When investment is socialised, the state is able to subordinate social production to social need, disciplining financial actors from engaging in the speculative tendencies amplified in private market exchange. When state capital is leading, economic policy determines not only the level of economic output, but the type of output produced in capitalist economies (Bellofiore 2020).

The intention in spelling out such an ambitious policy prescription is not to draw out unactionable implications beyond the scope of political possibility, for what is implied is a reformist agenda that the macro-financial architecture in polities that have embraced decades of private-led growth may be incapable of supporting. Despite tentative signs of a genuine embrace of active fiscal policy (van ‘t Klooster 2022, Schneider 2023), it is also clear that these are contested reforms, challenging the extent to which more radical forms of socialised investment can be realised.

Nor is it to demonstrate the superiority of the Chinese model where the ambitious state financing that has underpinned China’s economic growth has been fuelled by wage suppression, co-optation of private capital and unsustainable levels of public debt. As some have argued, China’s public investment-driven model of development has led to unproductive growth in real estate, wasteful infrastructure projects and surplus industrial capacity that has flooded global markets (Klein and Pettis 2021), although such observations ought still to be placed in the context of the global underinvestment gap. Green technologies alone demand an annual average of USD 5 trillion to meet the global target of net zero emission by 2050, yet Chinese investments, which accounted for nearly half of the global total of USD 499 billion in 2022, fall woefully short (World Energy Transitions Outlook 2023).

Rather, the article makes the case for the existence of counterfactual forms of economic governance in which states might harness the private sector to finance initiatives within the purview of public purpose. Corporate profits have soared in the wake of the pandemic so the problem is not one of financial paucity (Graham 2023). What may be required is a radical political and ideological reimagining of how industrial development should be financed. The Minsky-Marxist critique of actually existing Keynesianism in the 1960s was as much political as it was theoretical. The idea of a gradual push toward full employment in which there was a role to play for the self-regulating efficiency of private-led markets was politically unviable given the entrenched interests of private capital that the state was only too willing to support via private-inducing public investment and socialised bailouts (Sweezy and Magdoff 1977). Despite embrace of reformist Keynesian policies in the contemporary era, de-risking private investment and consistent big bang bailouts of the financial sector are oddly reminiscent of the failures of post-war Keynesianism. If the condition to subordinate finance to social need is a socialised investment, then something akin to revolutionary politics is needed to unmoor the entrenched interests of private power upon which OECD states have become increasingly dependent.

## Notes

1 The terms VC and PE are used interchangeably here since it is equity capital that the PE firms have raised from public investment, as opposed to debt leveraged buyout that traditionally marks the distinction between the two (Kuebart 2019).

2 Informed consent was obtained as per the research plan approved by the Ethical Review Committee Inner City Faculties, Maastricht University (ERCIC_129_01_04_2019). All personal identifying information has been anonymised.

3 Interview, former director, ISIF, 8th November, 2021.

4 Interview, former director, ISIF, 8th November, 2021.

5 Interview, former director, ISIF, 8th November, 2021.

6 Interview, director, ISIF, 8th November, 2021.

7 Interview, senior executive, SFPIM, 20 October 2021.

8 Interview, senior executive, SFPIM, 20 October 2021.
